# Hibernoma of the Upper Extremity: Complete Case of a Rare but Benign Soft Tissue Tumor

**DOI:** 10.1155/2019/6840693

**Published:** 2019-05-21

**Authors:** Thomas Reichel, Kilian Rueckl, Annabel Fenwick, Niklas Vogt, Maximilian Rudert, Piet Plumhoff

**Affiliations:** ^1^Department of Orthopedic Surgery, Koenig-Ludwig-Haus, University of Wuerzburg, Brettreichstraße 11, 97074 Wuerzburg, Germany; ^2^Department of Trauma Surgery, Klinikum Augsburg, 86156 Augsburg, Germany; ^3^Department of Pathology, University of Wuerzburg, 97080 Wuerzburg, Germany

## Abstract

Hibernoma is a rare benign lipomatous tumor showing differentiation of brown fatty tissue. To the author's best knowledge, there is no known case of malignant transformation or metastasis. Due to their slow, noninfiltrating growth hibernomas are often an incidental finding in the third or fourth decade of life. The vast majority are located in the thigh, neck, and periscapular region. A diagnostic workup includes ultrasound and contrast-enhanced MRI. Differential diagnosis is benign lipoma, well-differentiated liposarcoma, and rhabdomyoma. An incisional biopsy followed by marginal resection of the tumor is the standard of care, and recurrence after complete resection is not reported. The current paper presents diagnostic and intraoperative findings of a hibernoma of the upper arm and reviews similar reports in the current literature.

## 1. Introduction

Fat-containing tumors are the most common soft tissue tumors, their prevalence increasing with age [1]. The distinction between benign and malignant and common and rare lipomatous tumors can be challenging when based only on clinical examination and imaging studies. Hibernoma is a rare tumor of the brown fat tissue with a good prognosis that has to be included in differential diagnosis to prevent false treatment.

## 2. Case Presentation

A 44-year-old patient presented in our clinic with ongoing shoulder and arm pain for 3 years. He had noticed a painless tumor with feeling of pressure in his upper right arm for some months. The patient was diagnosed with an impingement syndrome and supraspinatus tendon rupture and consequently attributed the swelling to a spontaneous rupture of the long head of the biceps tendon. There was no history of recent trauma or injuries. The patient was in good health without systemic signs of infection, weight loss, or fever and stated no prior medical history.

Clinical examination showed a painless tumor on the right upper arm, starting lateral to the axillary fold expanding about 10 cm distally. On palpation, the tumor was elastic and firm with only little translational mobility. There was no sensory or motor dysfunction. The overlying skin showed no signs of infection or other noticeable changes.

## 3. Diagnostic Workup with Differential Diagnosis

Conventional X-rays revealed a soft tissue tumor without calcification. Ultrasound could confirm the suspected tumor, with clear margins to the surrounding muscle and similarities to fatty tissue. Slightly increased perfusion was present at the margins of the lesion.

**Figure 1:**
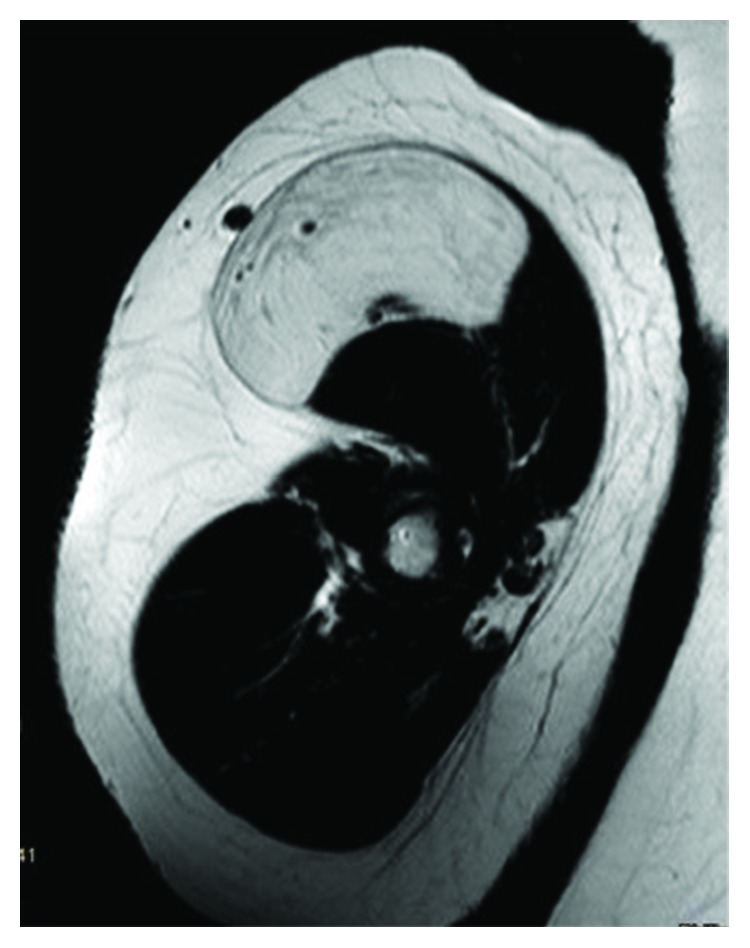
Axial MRI image, contrast-enhanced, T1w.

**Figure 2:**
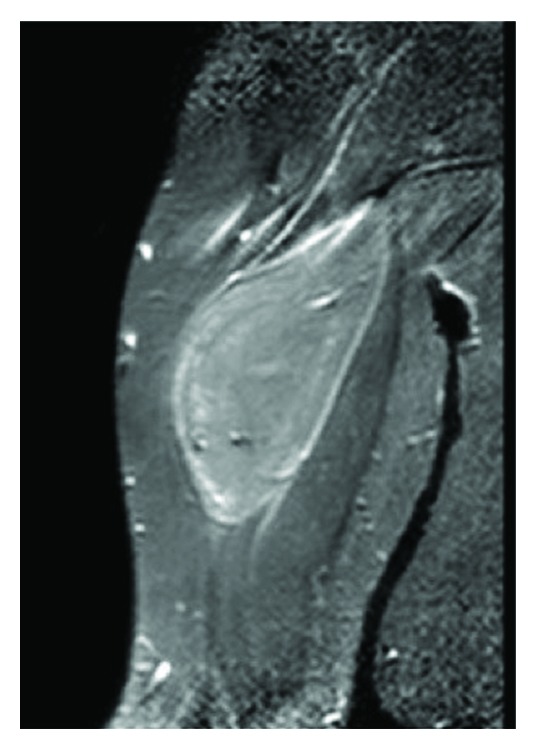
Coronal MRI image, contrast-enhanced, T2w.

**Figure 3:**
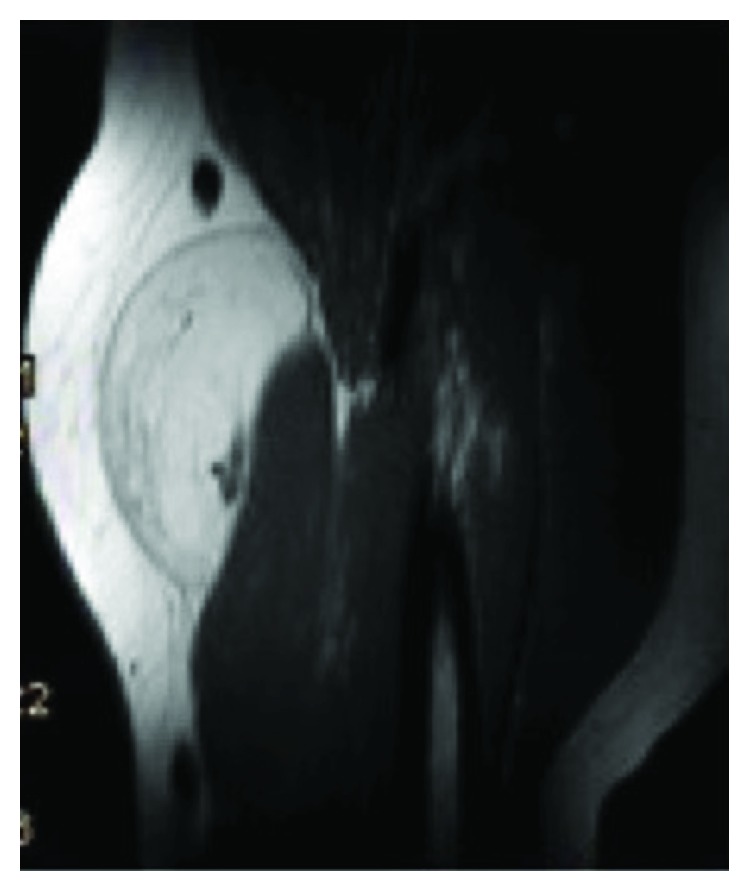
Sagittal MRI image, contrast-enhanced, T1w.

Contrast-enhanced MRI was performed. The tumor measured about 90 × 70 × 30 *mm* (length, width, and depth) with an intramuscular location, deep to the fascia of the M. biceps brachii. The fascia was not penetrated, and the tumor showed clear margins to the displaced muscle tissue. Signals in T1w and T2w were hyperintense but slightly inferior to subcutaneous fat on T1w. Contrast enhancement was shown in the periphery of the lesion (Figures 1–3).

The case was presented to the interdisciplinary tumor conference, and an incisional biopsy was scheduled to rule out malignancy.

## 4. Histopathological Findings

**Figure 4:**
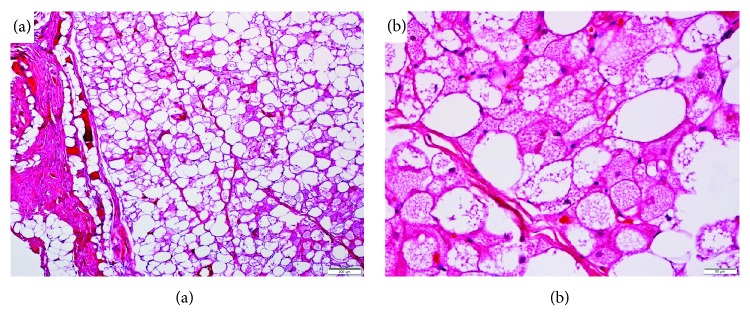
Histology of the tumor tissue. Multivacuolated, granular cytoplasma with small central nuclei and some univacuolar adipocytes. (a) 200 *μ*m and (b) 50 *μ*m.

Pathology showed multivacuolated, granular cytoplasma with small central nuclei, a characteristic for brown fat. Some univacuolar adipocytes were present. No atypical nuclei or high mitotic activity was seen (Figure 4). To rule out atypical lipomatous tumor, FISH was performed and showed no amplification of MDM2 gene. Some cells showed tri- to tetrasomal genome. Based on these findings, intramuscular hibernoma was diagnosed.

## 5. Therapy

**Figure 5:**
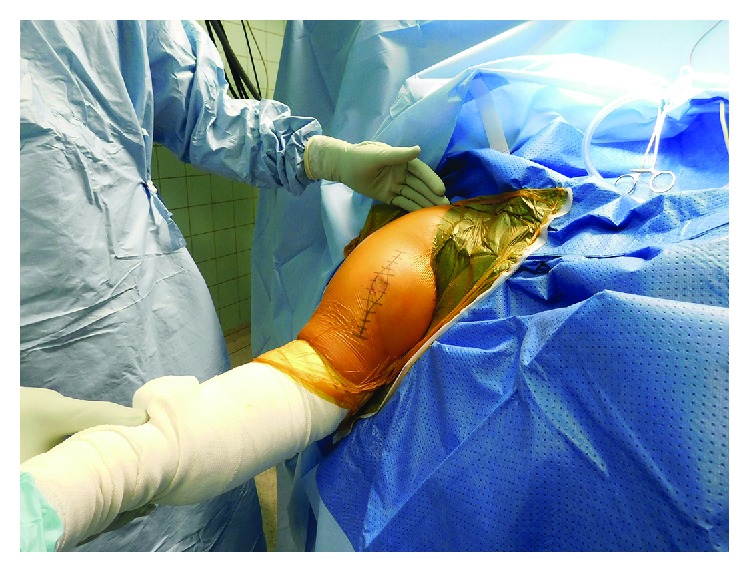
Surgical approach with excision of the site of biopsy, beach chair position.

**Figure 6:**
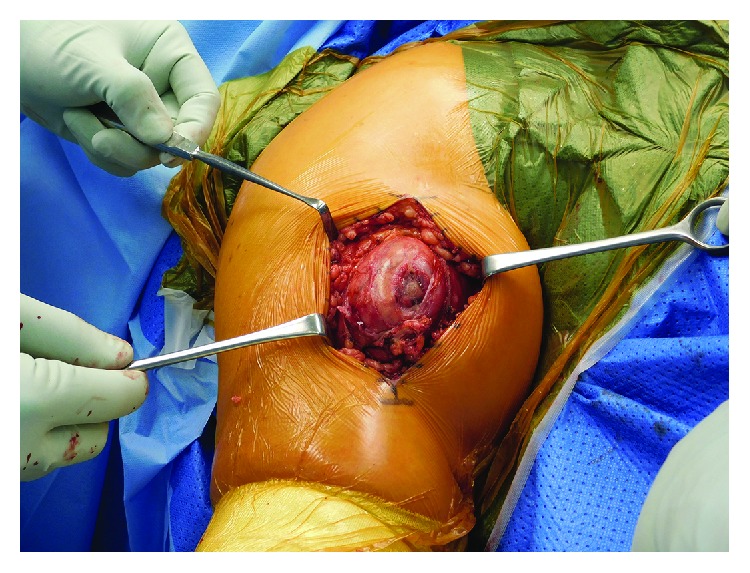
Intraoperative tumor appearance with small hematoma at the site of biopsy.

**Figure 7:**
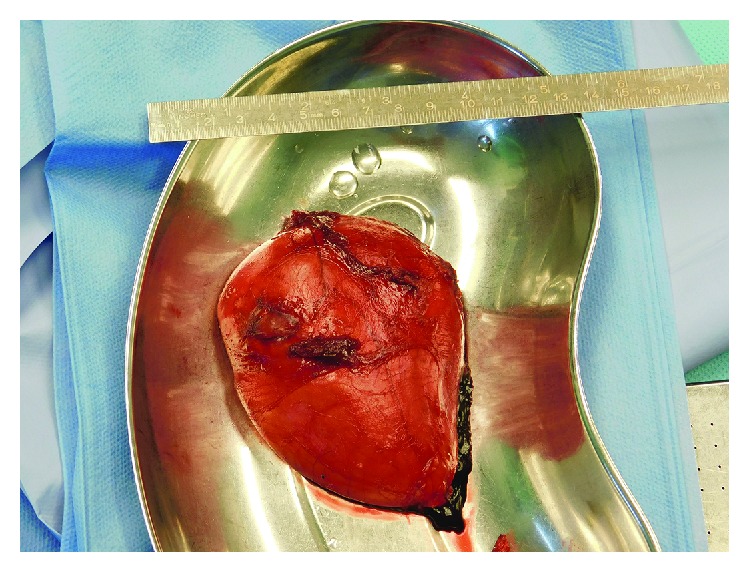
Removed tumor with marking sutures.

**Figure 8:**
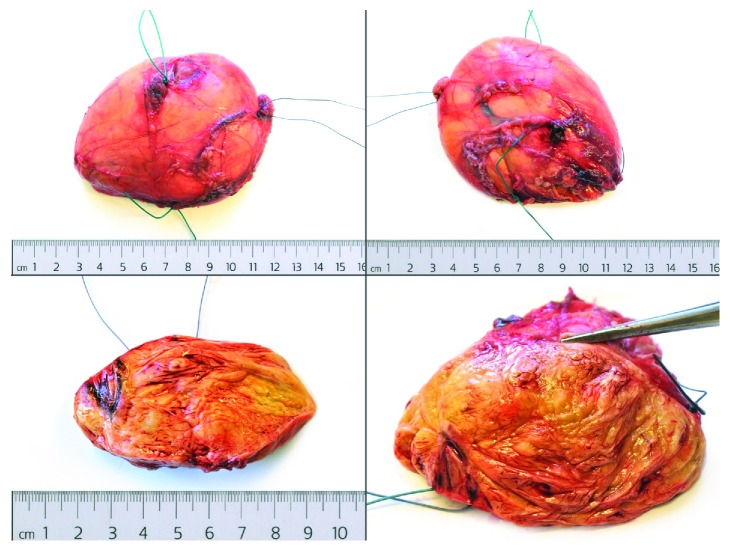
Gross appearance before histopathological workup.

**Figure 9:**
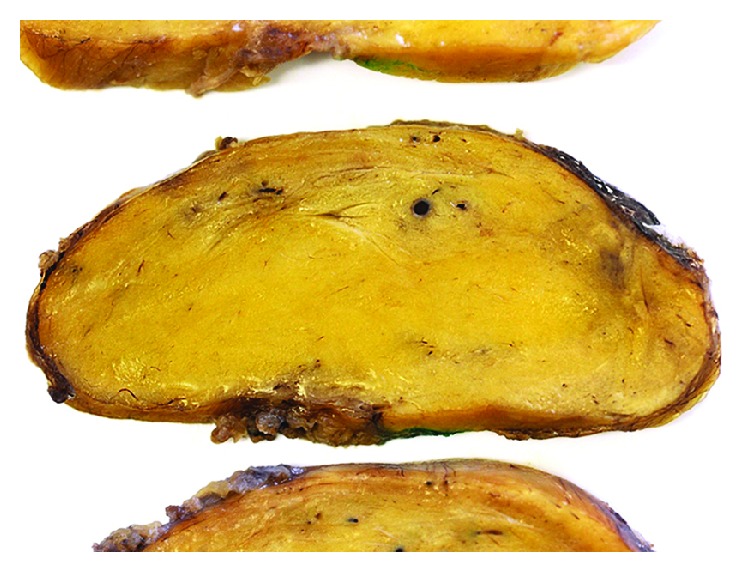
Cross-sectional view of the tumor.

After the incisional biopsy confirmed the diagnosis of a hibernoma, the patient was scheduled for tumor resection due to local symptoms. Marginal resection was performed (Figures 5–7) and showed a 110 g heavy tumor with well-defined margins, a surrounding thin capsule, and a brown fatty matrix corresponding to the previous biopsy (Figures 8 and 9).

## 6. Histopathological Findings after Tumor Removal

Diagnosis of hibernoma and its complete resection were confirmed. In follow-up MRIs, the patient showed no signs of complications or recurrence, although a long-term follow-up is needed because of the slow growing nature of the lesion.

## 7. Discussion and Literature Review

While lipomatous soft tissue tumors are frequently found incidentally, hibernoma is a very rare entity constituting only about 1% of benign lipomatous tumors, their real prevalence unknown [2]. In 1906, Merkel was the first to describe this tumor as “pseudolipoma” [3]. In 1914, the similarities between the glands of hibernating animals and this tumor were recognized by Gery and lead to the description of hibernoma [4]. There is only a very low number of a few hundred cases that have been published worldwide about this tumor with only a few publications constituting a high number of cases in a single institution [2, 5].

There are no known risk factors for hibernoma. As remnant tissue of brown fat, it usually occurs in areas like the neck, shoulder, and periscapular region while less frequently the trunk or retroperitoneum [5, 6]. Other occasional locations are the lower and upper extremities. The literature also reports rare locations, for example, intraosseous, perirenal, periadrenal, peripancreatic, paraortal, and intracranial [7]. Multiple lesions in a single patient are possible [8].

Growth is usually slow, and symptoms, if any, develop mostly in bigger tumors that compress neurovascular structures or irritate local tissue.

Imaging studies usually show characteristics of a fatty soft tissue tumor. Plain radiographs can display radiolucent areas without calcification or osseous abnormalities [9]. Ultrasound imaging is often unspecific in regard to other soft tissue tumors. Duplex ultrasound and angiography may reveal high perfusion, sometimes with arteriovenous shunts, but cannot further distinguish hibernoma from other tumors [10–13]. MRI usually shows a well-demarcated mass with hyperintense signal in T2w and intermediate signal in T1w between the signal of muscle or subcutaneous fat. Prominent low-signal septa can be seen. Intravenous gadolinium contrast can show variable, sometimes intense, contrast enhancement. Imaging characteristics vary because the different subtypes of hibernoma contain variable amounts of fat and water. Depending on location, size, and signal characteristics, differentiation to lipoma, well-differentiated liposarcoma, atypical lipomatous tumor, myxoid liposarcoma, or other malignant fatty tumors can be difficult to impossible [14]. PET-CT shows a medium to very high uptake of 18F-FDG as a result of high metabolic activity of brown fat tissue, with standard uptake values (SUV) similar or higher than, for example, liposarcoma [9, 13, 15, 16]. The only distinct characteristic of hibernoma is a fluctuating SUV over time because of the changing metabolic activity in brown fat cells, which is possibly even triggered by external temperature [14, 16]. The distinct sensitivity and specificity of these characteristics to exclude malignant lipomatous tumors are still unknown.

In the present case, after MRI imaging, atypical lipoma was suspected, but an incisional biopsy was performed to rule out liposarcoma because of size, location, and symptoms stated by the patient.

Histopathology showed multivacuolated fat cells with small nuclei. There are some described variabilities in appearance and staining characteristics, leading to different subcategories (typical, mixed, myxoid, lipoma-like, and spindle cell) that seem to have different prevalences depending on anatomic location, age, and gender. This variety in histological characteristics causes the aforementioned difficulty to distinguish hibernoma from lipoma and liposarcoma in imaging studies [2, 17, 18]. Microscopic and immunohistochemic characteristics usually suffice to diagnose hibernoma although it can be mistakenly diagnosed as a malignant lesion [19].

Treatment is complete marginal resection [2, 7]. The benign nature of hibernoma was questioned in some early reports [20, 21]. Current literature supports the belief that hibernoma is benign without reports of malignant transformation or metastatic spread [2, 5, 7].

Removal should be advised to rule out the possibility of a malignant lesion with hibernoma-like differentiation, which could be missed in a small biopsy [5]. Additionally, if patients present with symptoms leading to the finding of a hibernoma, marginal resection should be performed. After complete resection, there is usually no risk of recurrence [2].

## 8. Conclusion

Hibernoma is a rare, benign, lipomatous soft tissue tumor. There is no known risk of malignant transformation or metastatic spread. Differentiation to malignant soft tissue tumors like low-grade liposarcoma can be difficult to impossible when based only on radiographic imaging, and an incisional biopsy is mandatory in most cases. Marginal resection is curative with no reported recurrences after complete resection.
